# Hepatoprotective effect of water extract from *Chrysanthemum indicum* L. flower

**DOI:** 10.1186/1749-8546-8-7

**Published:** 2013-04-04

**Authors:** Sang Chul Jeong, Sang Min Kim, Yong Tae Jeong, Chi Hyun Song

**Affiliations:** 1 Department of Biotechnology, Daegu University, Gyeongsan, Gyeoongbuk 712-714, Republic of Korea

## Abstract

**Background:**

*Chrysanthemum indicum* L. flower (CIF) has been widely used as tea in Korea. This study aims to investigate the hepatoprotective effect of the hot water extract of CIF (HCIF) in *in vitro* and *in vivo* systems.

**Methods:**

Hepatoprotective activities were evaluated at 250 to 1000 μg/mL concentrations by an *in vitro* assay using normal human hepatocytes (Chang cell) and hepatocellular carcinoma cells (HepG2) against CCl_4_-induced cytotoxicity. Cytochrome P450 2E1, which is a key indicator of hepatic injury, was detected by western blot analysis using rabbit polyclonal anti-human CYP2E1 antibody. An *in vivo* hepatoprotective activity assay was performed at 1000 to 4000 μg/mL concentrations on CCl_4_-induced acute toxicity in rats, and the serum levels of glutamic oxaloacetic transaminase (GOT), glutamic pyruvic transaminase (GPT), alkaline phosphatase (ALP) and lactate dehydrogenase (LDH) were determined by standard enzyme assays.

**Results:**

The hepatoprotective effects of HCIF significantly reduced the levels of GOT (60.1%, *P* = 0.000) and GPT (64.5%, *P* = 0.000) compared with the vehicle control group (CCl_4_ alone). The survival rates of HepG2 and Chang cells were significantly improved compared with the control group [82.1% (*P* = 0.034) and 62.3% (*P* = 0.002), respectively]. HCIF [50 mg/kg body weight (BW)] treatment significantly reduced the serum levels of GOT (49.5%, *P* = 0.00), GPT (55.5%, *P* = 0.00), ALP (30.8%, *P* = 0.000) and LDH (45.6%, *P* = 0.000) compared with the control group in this *in vivo* study. The expression level of cytochrome P450 2E1 (CYP2E1) protein was also significantly decreased at the same concentration (50 mg/kg BW; *P* = 0.018).

**Conclusion:**

HCIF inhibited bioactivation of CCl_4_-induced hepatotoxicity and downregulates CYP2E1 expression *in vitro* and *in vivo.*

## Background

The liver is the major organ for the metabolism of xenobiotics and drugs. CCl_4_ is a widely used chemical and causes severe liver tissue damage by undergoing biotransformation by the cytochrome P450 system into a trichloromethyl free radical (CCl_3_^•^) and transformation into a highly reactive trichloromethylperoxy free radical (CCl_3_O_2_^•^). The resulting free radical damages liver cell membranes and organelles and causes swelling, necrosis of hepatocytes and the release of cytosolic enzymes, such as glutamic oxaloacetic transaminase (GOT), glutamic pyruvic transaminase (GPT), alkaline phosphatase (ALP) and lactate dehydrogenase (LDH), into serum and eventually kills cells [1-3]. Oriental traditional medicine has used the aerial parts (stem, leaves and flowers) of *Chrysanthemum indicum* to treat hypertensive symptoms and several infectious diseases, such as fever and stomatitis [4]. Notably, its flowers, which are used as traditional tea in Korea and China [5], are widely considered to have health benefits. Therefore, we investigated *C. indicum* L. flowers in this study. *Chrysanthemum indicum* L. flower (CIF) is a wild herb and has a long history of use as a traditional medicine, mainly for the treatment of inflammation, hypertension and respiratory diseases in Korean and Chinese medicine [6-9]. Several studies have demonstrated that the water extract of *C. indicum* L. has strong antioxidant effects and inhibitory effects against bacteria and viruses [10,11]. In addition, the methanol extract shows inhibitory activity of xanthine oxidase [12]. Several chemical compounds isolated from CIF exhibit inhibitory activity against nitric oxide (NO) in lipopolysaccharide-activated macrophages and rat lens aldose reductase [13].

The suppression of cytochrome P450 could result in reduced levels of reactive metabolites from xenobiotic exposure, decreasing liver injury [2,14]. Although several cytochrome P450 isoforms may metabolize CCl_4_, the cytochrome P450 2E1 (CYP2E1) isoform, which is ethanol inducible [15-17], has been widely studied. Altering expression of CYP2E1 activity affects susceptibility to hepatic injury from CCl_4_[18,19]. The expression of individual cytochrome P450 enzymes is regulated by both endogenous factors and foreign compounds, including drugs and natural compounds [20]. Natural compounds that reduce such bioactivating enzymes could be considered protective candidates against chemically induced toxicity, and CYP2E1 is well recognized for its role in the activation of many chemicals resulting in toxic and carcinogenic effects.

To our knowledge, no study has been conducted to determine the hepatoprotective effect of *C. indicum* L. against CCl_4_-induced toxicity. This study aims to investigate the hepatoprotective effect of HCIF in *in vitro* and *in vivo* systems.

## Methods

### Chemicals and reagents

Bovine serum albumin (BSA), 3-(4,5-dimethylthiazol-2-yl)-2,5-diphenyltetrazolium bromide (MTT), dimethyl sulfoxide (DMSO), sodium bicarbonate, silymarin and CCl_4_ were purchased from Sigma Chemical Co. (St. Louis, MO, USA). Fetal bovine serum (FBS), RPMI 1640 medium, Dulbecco’s modified Eagle’s medium (DMEM), trypsin-ethylenediaminetetraacetic acid (EDTA), penicillin and streptomycin were purchased from GIBCO BRL (Grand Island, NY, USA). GOT, GPT, ALP and LDH assay kits were purchased from Asan Pharmacology Co. (Seoul, Korea). Rabbit polyclonal anti-human CYP2E1 antibody was purchased from Chemicon International Inc. (Temecula, CA, USA). Goat polyclonal anti-human β-actin antibody, anti-rabbit IgG and anti-goat IgG were supplied by Santa Cruz Biotechnology (Santa Cruz, CA, USA).

### Preparation of hot water extract of CIF (HCIF)

CIF was obtained from the Daegu traditional medicine market (Seoul, Korea) and authenticated based on its microscopic and macroscopic characteristics by a local botany expert (Dr. Yang, Director, The Research Center for Resource of Oriental Medicine). CIFs (100 g) were ground into powder and decocted with distilled water (1 L) for 2 h. The decoction was collected twice, filtered (filter paper pore size, 0.45 μm) and lyophilized to obtain the HCIF. The HCIF was dissolved in saline for oral administration to rats.

### Cell cultures and viability

Hepatocellular carcinoma HepG2 (KCLB 88065) and normal human hepatocyte Chang (ATCC CCL-13) cell lines were obtained from the Korean Cell Line Bank (KCLB, Seoul, Korea) and American Type Culture Collection (ATCC, Manassas, VA, USA), respectively. The HepG2 and Chang cells were grown in RPMI-1640 and DMEM supplemented with 10% FBS, streptomycin (100 U/mL), penicillin (100 μg/mL) and sodium bicarbonate (3.7 g/mL). The cultures were maintained in 100-mm dishes at 37°C in a 5% CO_2_ humidified incubator (3111, ThermoForma, Ohio, USA). The cell viabilities of HCIF in HepG2 and Chang cells damaged by CCl_4_ were measured by the MTT assay. Briefly, cells were plated at a density of 2 × 10^5^ cells per well in a 96-well flat-bottom microtiter plate at three concentrations (250, 500 and 1000 μg/mL) of HCIF. After a 24-h incubation, the culture media were replaced with media containing CCl_4_ (8 mM) and incubated for 2 h. At the end of the incubation, 25 μL of MTT solution (5.0 mg/mL) was added to each well and incubated for 4 h at 37°C. The cells were then lysed with DMSO (200 μL per well), and the reduced intracellular formazan product was quantified in a Bio-Rad enzyme-linked immunosorbent assay microplate reader (680, Bio-Rad, Hercules, CA, USA) at 540 nm. Cell viability was expressed as the percentage of control absorbance at 540 nm. The data are presented as the mean of triplicate samples ± SD. Silymarin was used as the positive control [21,22].

### Animals

Male Sprague Dawley rats were purchased from Koatech Laboratory Animal Inc. (Seoul, Korea) and kept for 1 week on a commercial diet under environmentally controlled conditions (room temperature 19-25°C, relative humidity 50-60%) with free access to food and water. A controlled 12 h light/12 h dark cycle was maintained. Rats weighing 180–230 g were used in the CCl_4_-induced hepatotoxicity study. Animal experiments were performed in accordance with procedures approved by the Ethics Committee for Animal Experimentation of the Korea Food Research Institute.

### Treatment of animals

Liver damage was induced in rats by a 1:1 (v:v) mixture of CCl_4_ and olive oil by oral gavage as described by previous reports [23-25]. Rats were randomly grouped into four groups of nine animals each. Group I (untreated) rats were treated with olive oil alone (1 mL/kg BW). Group II (control) rats were treated with CCl_4_:olive oil (1 mL/kg BW). Group III (positive control) rats were pretreated with silymarin (50 mg/kg BW), and groups IV and V rats were pretreated with HCIF at the level of 50 or 100 mg/kg BW by oral gavage daily for 7 days before treatment with CCl_4_:olive oil (1:1).

### Enzymatic analysis

The cells were washed with phosphate-buffered saline (PBS) and exposed to fresh medium containing CCl_4_ (100 mM) at three concentrations (1, 2 and 4 mg/mL) of HCIF or medium alone. After 6 h of CCl_4_ treatment, GOT and GPT levels in the medium were measured as described in the assay kits. After removal of the medium, cells were washed twice with ice-cold PBS and used for western blot analysis.

In the animal experiment, all rats were anesthetized with ether 24 h after dosing with CCl_4_, and blood was then collected via the carotid artery. Plasma samples were collected from heparinized blood after centrifugation (Combi-514R, Hanil, Seoul, Korea) at 1,518 × g for 10 min at 4°C. The GOT, GPT and LDH levels were measured according to standard methods [26], and serum ALP was estimated by the Kind and Kings method [27].

### Western blot analysis of CYP2E1

After treatment with CCl_4_, the cells were washed twice with cold PBS and detached with 0.02% EDTA solution. Subsequently, the cells were treated with IPH lysis buffer and centrifuged at 14,240 × g for 20 min at 4°C. The cells were homogenized in buffer (pH 8.0) containing 50 mM Tris–HCl, 150 mM NaCl, 0.02% NaN_3_, 100 μg/mL phenylmethylsulfonyl fluoride (PMSF), 1 mg/mL aprotinin and 1% Triton X-100. Protein concentration was determined by the Bradford protein assay kit (Bio-Rad). Twenty micrograms samples of total cell lysates were size fractionated by SDS-PAGE and electrophoretically transferred to nitrocellulose membranes by a Hoefer electro transfer system (Amersham Pharmacia Biotech Inc., NJ, USA). The membranes were incubated overnight with blocking buffer containing 10 mM Tris–HCl, 150 mM NaCl, 0.1% Tween 20 and nonfat dry milk at 4°C. The membranes were then incubated for 2 h at room temperature with 1:1000 diluted primary antibodies (rabbit polyclonal anti-human CYP2E1 antibody and goat polyclonal anti-human β-actin antibody). After washing with blocking buffer 3 times for 10 min, membranes were probed with 1:2000 diluted secondary antibodies (horseradish peroxidase-linked anti-rabbit, anti-goat IgG) for 1 h, washed 3 times for 10 min and developed with an ECL western blotting detection system (Amersham Pharmacia Biotech Inc.).

### Histopathological examination

Fresh liver tissues, previously trimmed to approximately 2-mm thickness, were placed in plastic cassettes and immersed in neutral buffered formalin for 24 h. Fixed tissues were processed routinely and then embedded in paraffin, sectioned, deparaffinized and rehydrated. The extent of CCl_4_-induced necrosis was evaluated by morphological changes in liver sections stained with hematoxylin and eosin (Axiolab reflected light microscope, Carl Zeiss, Germany).

### Statistical analysis

The results are presented as the mean ± SD (calculated from n = 3 and n = 9 in the *in vitro* and *in vivo* studies, respectively). The significance of differences among groups of data was determined using SPSS 18.0 for Windows (IBM, Chicago, IL, USA). Student’s t-test was used to compare two independent groups. Statistical significance was accepted for *P* values of < 0.05.

## Results

### Effect of HCIF on CCl_4_-induced hepatotoxicity *in vitro*

**Figure 1:**
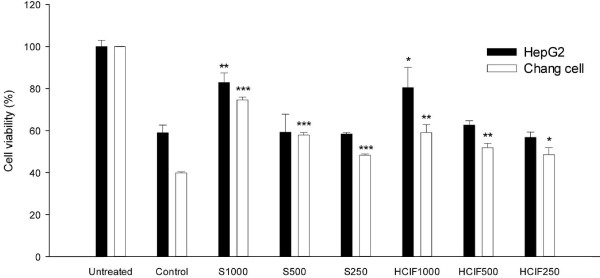
**Protective effect of the *Chrysanthemum indicum *L. flower hot water extract (HCIF) against CCl**_**4**_**-induced cytotoxicity in a hepatocyte cell line.** Untreated, cells alone; Control, cells + CCl_4_; S, cell + CCl_4_ + silymarin; HCIF, cell + CCl_4_ + HCIF. ^*^P < 0.05, ^**^P < 0.01 and ^***^P < 0.001, significantly different from the control group.

The 8 mM CCl_4_-exposed HepG2 and Chang cells exhibited cell viabilities of 58% and 39%, respectively, compared with untreated controls (Figure 1). Viability of these CCl_4_-exposed cells was exhibited in a dose-dependent manner when pretreated with various HCIF concentrations. The percentage viability of HCIF + CCl_4_ was less than the silymarin + CCl_4_, which produced 82% cell viability (*P* = 0.034) at a dose of 8 mM compared with the CCl_4_-treated control group.

**Figure 2:**
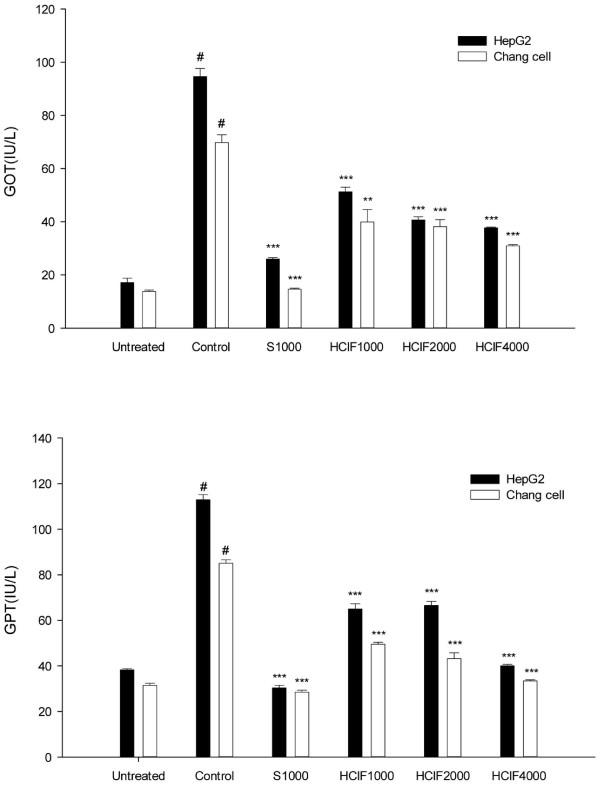
**Effect of HCIF on GOT and GPT leakage in a hepatocyte cell line.** Untreated, cells alone; Control, cells + CCl_4_; S, cell + CCl_4_ + silymarin (1 mg/mL); HCIF, exposed to cell + CCl_4_ + CIF (1, 2 and 4 mg/mL). ^*^P < 0.05, ^**^P < 0.01 and ^***^P < 0.001, significantly different from the control group. ^#^*P* < 0.001, significantly different from the untreated group.

CCl_4_-induced hepatocyte cell lines expressed high levels of GOT and GPT as shown in Figure 2. However, GOT (39.8 IU/L) and GPT (44.3 IU/L) levels were reduced in the 4 mg/mL HCIF-treated HepG2 cells and significantly reduced by 60.1% (*P* = 0.000) and 64.5% (*P* = 0.000), respectively, compared with the control group. Likewise, HCIF effectively and significantly lowered levels of GPT (33.4 IU/L; *P* = 0.000) and GOT (34.2 IU/L; *P* = 0.002) in Chang cells. Silymarin also caused a significant reduction in GOT and GPT leakage (*P* = 0.000) at 4 mg/mL HCIF.

### Effect of HCIF on CCl_4_-induced hepatotoxicity *in vivo*

CCl_4_ treatment caused a significant elevation of serum GOT, GPT, ALP and LDH activities (5-, 10-, 2- and 3.5-fold, respectively) in rats. These elevated activities were significantly decreased by 50 mg/kg BW HCIF treatment [49.5% (*P* = 0.000), 55.5% (*P* = 0.000), 30.8% (*P* = 0.000) and 45.6% (*P* = 0.000), respectively]. Silymarin also significantly reduced the CCl_4_-induced elevation of serum enzymatic activities at 50 mg/kg BW concentration (*P* = 0.000). In the CCl_4_-induced acute hepatitis model (Table 1), inhibitory effects of HCIF on the release of GOT and GPT into rat serum were similar to or lower than the corresponding effects mediated by silymarin (50 mg/kg BW). The reduction of GOT, GPT, ALP and LDH levels after administration of HCIF could indicate the stabilization of the plasma membrane in liver and repair of hepatic tissue damage caused by CCl_4_.

**Table 1:** **Hepatoprotective effect of HCIF on CCl**_**4**_**-induced toxicity in rats**

	**GOT (IU/L)**	**GPT (IU/L)**	**ALP (IU/L)**	**LDH (IU/L)**
Untreated	41.4 ± 5.9	14.2 ± 0.4	161.6 ± 16.7	720.4 ± 51.1
CCl_4_-treated control	197.3 ± 10.4^#^	148.6 ± 9.6^#^	330.5 ± 36.3^#^	2516.2 ± 439.4^#^
Silymarin + CCl_4_ (50 mg/kg)	71.5 ± 4.9^***^	60.5 ± 6.8^***^	202.3 ± 34.2^***^	1122.1 ± 135.5^***^
HCIF50 + CCl_4_ (50 mg/kg)	99.5 ± 7.8^***^	66.1 ± 14.0^***^	228.7 ± 26.3^***^	1368.6 ± 144.3^***^

Serum GOT, GPT, ALP and LDH levels were determined by commercial kits. Each value is the mean ± SD; n = 9 rats. ^#^*P* < 0.001, significantly different from the untreated group. ^***^P < 0.01, significantly different from the CCl_4_-treated control group.

### Effect of HCIF on CYP2E1 expression

**Figure 3:**
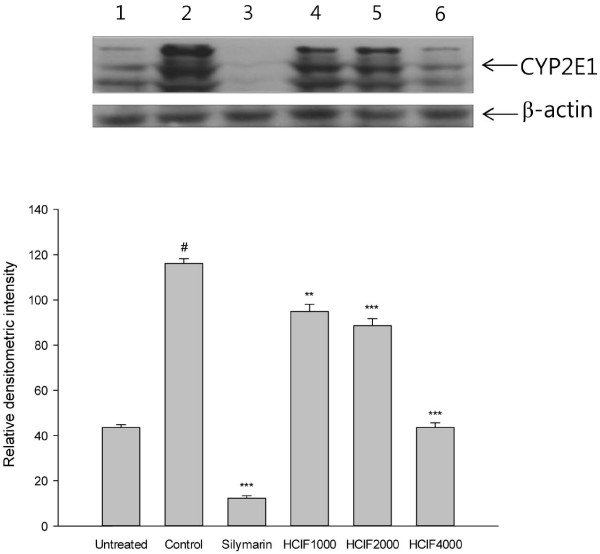
**Western blot analysis of CYP2E1 levels in Chang cell line.** The expression level of CYP2E1 protein was detected by western blot analysis with anti-human CYP2E1 antibody. Chang cells were treated with saline (lane 1, untreated), CCl_4_ (lane 2, control), CCl_4_ + silymarin 1 mg/mL (lane 3), CCl_4_ + HCIF 1 mg/mL (lane 4), CCl_4_ + HCIF 2 mg/mL (lane 5) and CCl_4_ + HCIF 4 mg/mL (lane 6). All bands were quantified by densitometric analysis. Each bar represents the mean ± SD replicated in triplicate. ^**^P < 0.01 and ^***^P < 0.001, significantly different from the control group. ^#^*P* < 0.001, significantly different from the untreated group.

**Figure 4:**
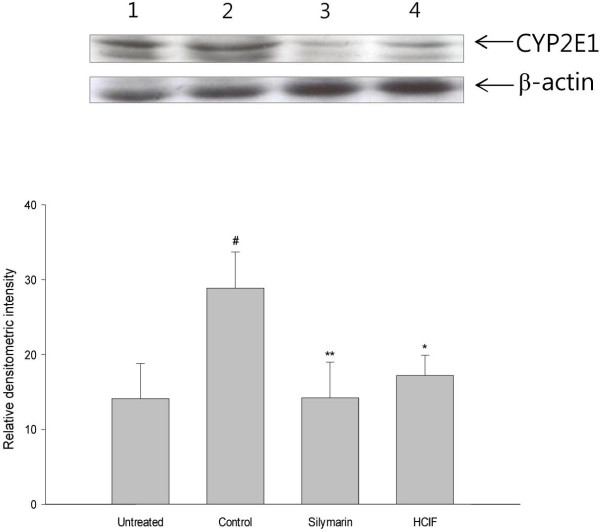
**Western blot analysis of CYP2E1 levels in rat liver.** The expression level of CYP2E1 was detected by western blot analysis of protein samples from rat liver. Rats were treated with olive oil (lane 1, untreated), CCl_4_ + saline (lane 2, control), CCl_4_ + silymarin 50 mg/kg BW (lane 3, silymarin) and CCl_4_ + CIF 50 mg/kg BW (lane 4, HCIF) for 7 days. All hepatotoxicity was induced in rats with 1 mg/kg body weight of CCl_4_. All bands were quantified by densitometric analysis. ^*^P < 0.05 and ^**^P < 0.01, significantly different from the control group. ^#^*P* < 0.01, significantly different from the untreated group.

Silymarin decreased CYP2E1 protein levels *in vitro* and *in vivo* (Figures 3 and 4, lane 3). CYP2E1 expression in Chang cells was suppressed by HCIF treatment in a dose-dependent manner (Figure 3, lanes 4–6). CYP2E1 levels were also reduced to 43.1% (*P* = 0.018) *in vivo* at a dose of 50 mg/kg BW (Figure 4, lane 4)*.*

### Histopathological examination

**Figure 5:**
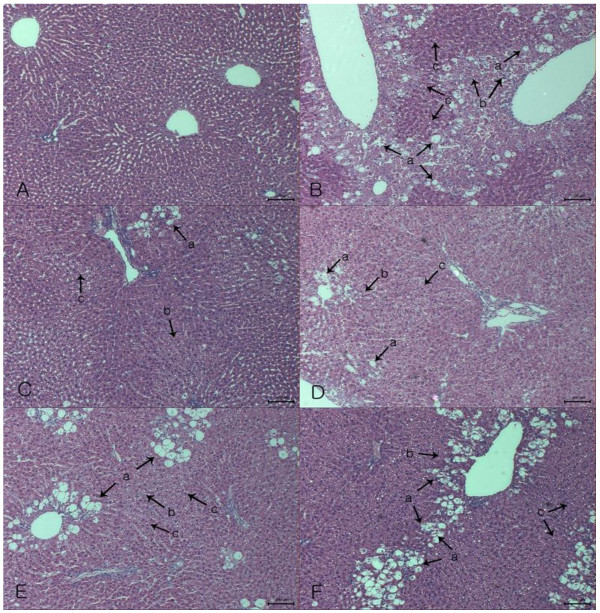
**Photomicrographs of paraffin-embedded rat liver.** Each sample was pretreated for 7 days and then treated with a single dose of CCl_4_. (**A**) untreated group (group I); (**B**) CCl_4_ alone (group II); (**C**) silymarin (50 mg/kg BW, group III); (**D**) HCIF (50 mg/kg BW, group IV); (**E**) HCIF (100 mg/kg BW, group V); (**F**) tea seed oil (200 mg/kg BW, group VI). All hepatotoxicity was induced in rats with 1 mg/kg body weight of CCl_4_ and analyzed 24 h later. Each of the arrows indicated vacuole formation (a), necrosis (b) and fatty degeneration (c).

We examined whether HCIF could affect anatomical changes in injured liver tissue. Photomicrographs of hematoxylin and eosin-stained liver tissue are shown in Figure 5. Histopathological changes were prominent compared with those in rats in the untreated (group I) and control (group II) groups. No histological abnormalities were observed of group I (Figure 5A). However, hepatocytes around the central vein revealed complete necrosis (arrow b) and loss of the cellular boundary (Figure 5B) in group II. Additionally, hepatic cells were found to have fatty degeneration (arrow c) and cytoplasmic vacuolization (arrow a). Numerous diffuse ballooning degeneration of different sizes and larger magnitude compared with group I was observed. Pretreatment of HCIF (Figure 5D and E) resulted in less severe histopathological alterations compared with group II. Furthermore, remarkable changes, such as less ballooning degeneration, cytoplasmic vacuolization and fatty degeneration, were observed in the CCl_4_ + HCIF-treated rat livers compared with that of group II. The numbers of CCl_4_-induced histopathological alterations were dramatically decreased in the HCIF-treated (group IV and V) and 50 mg/kg silymarin treatment (group III) groups.

## Discussion

Water extracts derived from many natural products possess hepatoprotective effects [28,29]. The hepatoprotective effects of HCIF were investigated in this study. CCl_4_-induced toxicity is commonly used to study the hepatoprotective effects of drugs or medicinal plant extracts using *in vivo* and *in vitro* techniques [14,30]. Usually, the extent of hepatic damage is assessed by histopathological examination and measurement of GOT, GPT and ALP levels released into serum [31,32]. This work demonstrated that HCIF significantly affected CCl_4_-induced hepatotoxicity in hepatocyte cell lines and rats. Recovery of normal serum levels of transaminases indicated healing of hepatic parenchyma and regeneration of hepatocytes [33]. In this study, enzyme levels significantly decreased to 49.5% and 55.5% at 50 mg/kg BW dose of HCIF, suggesting that HCIF has a potent hepatoprotective effect on CCl_4_-treated rats. GOT and GPT levels in hepatocytes in this cell culture study were comparable to *in vivo* results.

The hepatocellular carcinoma cell line HepG2 is a reliable model that is easy to culture, well characterized and widely used for biochemical and drug toxicity studies. HepG2 cells possess many morphological and biochemical features of normal hepatocytes, and many hepatoprotective compounds have been studied using HepG2 cells [34-38]. Silymarin or its main ingredient silibinin can inhibit cancer cells [39]. In this study, silymarin increased cell viability resulting from CCl_4_-induced hepatotoxicity. The mechanism of CCl_4_-induced damage involves the biotransformation of CCl_4_ into a highly reactive trichloromethyl free radical (CCl_3_^•^). Silymarin is a new hepatoprotective agent [40], which scavenges radicals, prevents glutathione (GSH) oxidation and depletion and stabilizes membranes [41-43]. Many previous reports have confirmed that many antioxidants decrease toxicity and lipid peroxidation induced by CCl_4_[44,45]. Shear *et al*. [42] studied HepG2 cell viability with silymarin, which increased HepG2 cell viability against the oxidative metabolite of acetaminophen. In the present study, we did not investigate whether HCIF has anticancer effects.

Western blotting was performed on total protein samples isolated from rat liver homogenates and Chang cells to assess CYP2E1 protein expression. CYP2E1 has been demonstrated to be largely responsible for the activation of CCl_4_ to its toxic metabolites [46], and pretreatment of rats with CYP2E1 inhibitors can protect against CCl_4_-induced hepatotoxicity [47]. We found decreased expression of CYP2E1 protein in HCIF-treated Chang cells (Figure 3) and hepatic microsomes in HCIF-treated rats (Figure 4)*.* The phytochemical profile of HCIF contains large amounts of caffeic acid, luteolin, kaempferol, flavonoids, terpenoids and phenolic compounds [13,48]. Polyphenols, which are strong antioxidants, prevent ethanol-induced CYP2E1 expression in HepG2 cells [49]. The downregulation of CYP2E1 expression decreases the formation of CCl_3_^•^ and reduces hepatocyte necrosis and hepatocellular injury [47]. Several previous studies have demonstrated that CCl_4_-induced hepatotoxicity could be modulated by substances that influence CYP2E1 activity [50,51]. In particular, compounds or drugs that induce CYP2E1 could potentiate the hepatic toxicity of CCl_4_[52,53]. Compounds that inhibit CYP2E1 could protect cells against CCl_4_-induced toxicity [22,46]. The induction or inhibition of CCl_4_ biotransformation may subsequently influence metabolic activation or detoxification of CCl_4_. Generally, CYP2E1 participates in the metabolism of small organic molecules, such as carbon tetrachloride, acetaminophen and nitrosamines [15,17,54]. Thus, CYP2E1 inhibition by HCIF not only protects cells against CCl_4_-induced hepatotoxicity, but also reduces xenobiotic toxicity.

CCl_4_ causes increased formation of pro-oxidants (trichloromethyl radical) and a concomitant decrease in the antioxidant status of the cell [55]. Overproduction of oxygen radicals causes an imbalance in oxidant-antioxidant capacity and increased attacks on unsaturated fatty acid of lipid structures leading to lipid peroxidation and damaging effects on proteins [56]. These pro-oxidant molecules attack microsomal lipids and form peroxidation products [57,58]. Changes in biochemical indices and histopathological appearance in CCl_4_-treated rats were significant when compared with the untreated group (group I). HCIF-pretreated rats showed a significant hepatoprotective effect of HCIF against CCl_4_-induced liver injury in rats. The histopathological appearance and biochemical indices of 50 mg/kg BW HCIF-pretreated rats were similar to that of the untreated group (group I). CCl_4_ treatment of rats markedly increased serum ALP and LDH levels, which reflect the severity of liver injury [59]. Large quantities of ALT and LDH secreted into serum may be associated with severe liver injury. As previously reported, CIF has a large amount of phenolic compounds, and the water extract of CIF exhibited high antioxidant activity [60]. Lipid peroxidation, the principal cause of CCl_4_-induced liver injury, is associated with the free-radical metabolite of CCl_4_. One of the hepatoprotective activities of HCIF may also result from its antioxidative properties.

## Conclusions

HCIF inhibited bioactivation of CCl_4_-induced hepatotoxicity and downregulated CYP2E1 expression *in vitro* and *in vivo.*

## Abbreviations

ALP: Alkaline phosphatase; BSA: Bovine serum albumin; BW: Body weight; CCl4: Carbon tetrachloride; CIF: *Chrysanthemum indicum* L. flower; CYP2E1: Cytochrome P450 2E1 protein; DMEM: Dulbecco’s modified Eagle’s medium; DMSO: Dimethyl sulfoxide; EDTA: Ethylenediaminetetraacetic acid; FBS: Fetal bovine serum; GOT: Glutamic oxaloacetic transaminase; GPT: Glutamic pyruvic transaminase; HCIF: Hot water extract of CIF; HepG2: Hepatocellular carcinoma cell line; LDH: Lactate dehydrogenase; MTT: 3-(4,5-dimethylthiazol-2-yl)-2,5-diphenyltetrazolium bromide; NO: Nitric oxide

## Competing interests

The authors declare that they have no competing interests.

## Author’s contributions

CHS, SCJ and SMK designed the study and wrote the manuscript. SCJ, SMK and YTJ performed the experiments. SCJ, SMK, CHS and YTJ analyzed the data. All authors read and approved the final manuscript.
